# Bacterial Vaginosis Associated with Increased Risk of Female-to-Male HIV-1 Transmission: A Prospective Cohort Analysis among African Couples

**DOI:** 10.1371/journal.pmed.1001251

**Published:** 2012-06-26

**Authors:** Craig R. Cohen, Jairam R. Lingappa, Jared M. Baeten, Musa O. Ngayo, Carol A. Spiegel, Ting Hong, Deborah Donnell, Connie Celum, Saidi Kapiga, Sinead Delany, Elizabeth A. Bukusi

**Affiliations:** 1 Department of Obstetrics, Gynecology and Reproductive Sciences, University of California San Francisco, San Francisco, United States of America; 2 Women's Health & Empowerment Center of Expertise, University of California Global Health Institute, San Francisco, United States of America; 3 Department of Global Health, University of Washington, Seattle, Washington, United States of America; 4 Department of Medicine, University of Washington, Seattle, Washington, United States of America; 5 Department of Pediatrics University of Washington, University of Washington, Seattle, Washington, United States of America; 6 Department of Epidemiology, University of Washington, Seattle, Washington, United States of America; 7 Center for Microbiology Research, Kenya Medical Research Institute, Nairobi, Kenya; 8 Department of Pathology and Laboratory Medicine, University of Wisconsin, Madison, United States of America; 9 Vaccine and Infectious Disease Division, Fred Hutchinson Cancer Research Center, Seattle, Washington, United States of America; 10 Department of Epidemiology & Population Health, London School of Hygiene and Tropical Medicine, London, United Kingdom; 11 Wits Reproductive Health & HIV Institute, University of Witwatersrand, Johannesburg, South Africa; NICHD, United States of America

## Abstract

**Background:**

Bacterial vaginosis (BV), a disruption of the normal vaginal flora, has been associated with a 60% increased risk of HIV-1 acquisition in women and higher concentration of HIV-1 RNA in the genital tract of HIV-1–infected women. However, whether BV, which is present in up to half of African HIV-1–infected women, is associated with an increase in HIV-1 transmission to male partners has not been assessed in previous studies.

**Methods and Findings:**

We assessed the association between BV on female-to-male HIV-1 transmission risk in a prospective study of 2,236 HIV-1–seropositive women and their HIV-1 uninfected male partners from seven African countries from a randomized placebo-controlled trial that enrolled heterosexual African adults who were seropositive for both HIV-1 and herpes simplex virus (HSV)-2, and their HIV-1–seronegative partners. Participants were followed for up to 24 months; every three months, vaginal swabs were obtained from female partners for Gram stain and male partners were tested for HIV-1. BV and normal vaginal flora were defined as a Nugent score of 7–10 and 0–3, respectively. To reduce misclassification, HIV-1 sequence analysis of viruses from seroconverters and their partners was performed to determine linkage of HIV-1 transmissions. Overall, 50 incident HIV-1 infections occurred in men in which the HIV-1–infected female partner had an evaluable vaginal Gram stain. HIV-1 incidence in men whose HIV-1–infected female partners had BV was 2.91 versus 0.76 per 100 person-years in men whose female partners had normal vaginal flora (hazard ratio 3.62, 95% CI 1.74–7.52). After controlling for sociodemographic factors, sexual behavior, male circumcision, sexually transmitted infections, pregnancy, and plasma HIV-1 RNA levels in female partners, BV was associated with a greater than 3-fold increased risk of female-to-male HIV-1 transmission (adjusted hazard ratio 3.17, 95% CI 1.37–7.33).

**Conclusions:**

This study identified an association between BV and increased risk of HIV-1 transmission to male partners. Several limitations may affect the generalizability of our results including: all participants underwent couples HIV counseling and testing and enrolled in an HIV-1 prevention trial, and index participants had a baseline CD4 count ≥250 cells/mm^3^ and were HSV-2 seropositive. Given the high prevalence of BV and the association of BV with increased risk of both female HIV-1 acquisition and transmission found in our study, if this association proves to be causal, BV could be responsible for a substantial proportion of new HIV-1 infections in Africa. Normalization of vaginal flora in HIV-1–infected women could mitigate female-to-male HIV-1 transmission.

*Trial Registration:* ClinicalTrials.com NCT00194519

*Please see later in the article for the Editors' Summary*

## Abstract 2

In a prospective study, Craig Cohen and colleagues investigate the association between bacterial vaginosis and the risk of female-to-male HIV-1 transmission.

## Editors' Summary

**Background:**

Since the first reported case of AIDS in 1981, the number of people infected with HIV, the virus that causes AIDS, has risen steadily. By the end of 2010, 34 million people were living with HIV/AIDS. At the beginning of the epidemic more men than women were infected with HIV. Now, however, 50% of all adults infected with HIV are women and in sub-Saharan Africa, where two-thirds of HIV-positive people live, women account for 59% of people living with HIV. Moreover, among 15–24 year-olds, women are eight times more likely than men to be HIV-positive. This pattern of infection has developed because most people in sub-Saharan Africa contract HIV through unprotected heterosexual sex. The risk of HIV transmission for both men and women in Africa and elsewhere can be reduced by abstaining from sex, by only having one or a few partners, by always using condoms, and by male circumcision. In addition, several studies suggest that antiretroviral therapy (ART) greatly reduces HIV transmission.

**Why Was This Study Done?:**

Unfortunately, in sub-Saharan Africa, only about a fifth of HIV-positive people are currently receiving ART, which means that there is an urgent need to find other effective ways to reduce HIV transmission in this region. In this prospective cohort study (a type of study that follows a group of people for some time to see which personal characteristics are associated with disease development), the researchers investigate whether bacterial vaginosis—a condition in which harmful bacteria disrupt the normal vaginal flora—increases the risk of female-to-male HIV transmission among African couples. Bacterial vaginosis, which is extremely common in sub-Saharan Africa, has been associated with an increased risk of HIV acquisition in women and induces viral replication and shedding in the vagina in HIV-positive women, which may mean that HIV-positive women with bacterial vaginosis are more likely to transmit HIV to their male partners than women without this condition. If this is the case, then interventions that reduce the incidence of bacterial vaginosis might be valuable HIV prevention strategies.

**What Did the Researchers Do and Find?:**

The researchers analyzed data collected from 2,236 heterosexual African couples enrolled in a clinical trial (the Partners in Prevention HSV/HIV Transmission Study) whose primary aim was to investigate whether suppression of herpes simplex virus infection could prevent HIV transmission. In all the couples, the woman was HIV-positive and the man was initially HIV-negative. The female partners were examined every three months for the presence of bacterial vaginosis and the male partners were tested regularly for HIV infection. The researchers also determined whether the men who became HIV-positive were infected with the same HIV strain as their partner to check that their infection had been acquired from this partner. The HIV incidence in men whose partners had bacterial vaginosis was 2.9 per 100 person-years (that is, 2.9 out of every 100 men became HIV-positive per year) whereas the HIV incidence in men whose partners had a normal vaginal flora was 0.76 per 100 person-years. After controlling for factors that might affect the risk of HIV transmission such as male circumcision and viral levels in female partner's blood, the researchers estimated that bacterial vaginosis was associated with a 3.17-fold increased risk of female-to-male HIV transmission in their study population.

**What Do These Findings Mean?:**

These findings suggest that HIV-positive African women with bacterial vaginosis are more than three times as likely to transmit HIV to their male partners as those with a normal vaginal flora. It is possible that some unknown characteristic of the men in this study might have increased both their own risk of HIV infection and their partner's risk of bacterial vaginosis. Nevertheless, because bacterial vaginosis is so common in Africa (half of the women in this study had bacterial vaginosis at least once during follow-up) and because this condition is associated with both female HIV acquisition and transmission, these findings suggest that bacterial vaginosis could be responsible for a substantial proportion of new HIV infections in Africa. Normalization of vaginal flora in HIV-infected women by frequent presumptive treatment with antimicrobials (treatment with a curative dose of antibiotics without testing for bacterial vaginosis) or possibly by treatment with probiotics (live “good” bacteria) might, therefore, reduce female-to-male HIV transmission in sub-Saharan Africa.

**Additional Information:**

Please access these Web sites via the online version of this summary at http://dx.doi.org/10.1371/journal.pmed.1001251.

- Information is available from the US National Institute of Allergy and infectious diseases on all aspects of HIV infection and AIDS and on bacterial vaginosis
- The US Centers for Disease Control and Prevention has information on all aspects of HIV/AIDS, including specific information about HIV/AIDS and women; it also has information on bacterial vaginosis (in English and Spanish)
- NAM/aidsmap provides basic information about HIV/AIDS, and summaries of recent research findings on HIV care and treatment, and information on bacterial vaginosis and HIV transmission (in several languages)
- Information is available from Avert, an international AIDS nonprofit group on many aspects of HIV/AIDS, including detailed information on HIV and AIDS prevention, on women, HIV and AIDS and on HIV/AIDS in Africa (in English and Spanish); personal stories of women living with HIV are available; the website Healthtalkonline also provides personal stories about living with HIV
- More information about the Partners in Prevention HSV/HIV Transmission Study is available

## Introduction

Worldwide, an estimated 33.3 million people are infected with HIV-1, 60% in sub-Saharan Africa, where women account for the majority of those infected [1]. Antiretroviral therapy (ART), through reducing HIV-1 plasma [2] and genital HIV-1 RNA concentrations [3], has been associated with >90% reduction in HIV-1 transmission in observational studies [4] and a recent trial of earlier ART initiation [5]. However, only about half of HIV-1–infected adults qualify for ART initiation per current country guidelines, and only 37% of those qualifying for ART in Africa received treatment [1]. Thus, new HIV-1 prevention strategies that will reduce HIV-1 risk for those not on ART remain an urgent need.

Bacterial vaginosis (BV) is a common disorder characterized by changes in vaginal flora in which normally predominant *Lactobacillus* species are replaced by potential pathogens including *Gardnerella vaginalis*, genital *Mycoplasma*, and fastidious anaerobic bacteria [6],[7]. For unknown reasons, BV is considerably more common among women in sub-Saharan Africa and other resource-poor countries than in developed countries, affecting up to 55% of women in some studies [8]–[10]. BV has been associated with a 60% increased risk of HIV-1 acquisition in women [11], and, among women with HIV-1, with higher HIV-1 concentrations in cervicovaginal fluids [12]–[14]. Bacteria associated with BV can induce viral replication and shedding in the genital tract [15],[16], which may lead to increased HIV-1 infectiousness for women with BV [17],[18]. However, to date, no study has examined whether BV increases the risk of female-to-male HIV-1 transmission. We hypothesized that HIV-1–infected women with BV have an increased risk of female-to-male HIV-1 transmission than women with normal vaginal flora. To answer this question, we prospectively studied a cohort of African heterosexual couples in which the female was HIV-1 seropositive and the male was HIV-1–seronegative who were enrolled in a randomized placebo-controlled trial of dually HIV-1 and herpes simplex virus (HSV) type 2–seropositive heterosexual African adults, and their HIV-1–seronegative partners.

## Methods

### Ethics Statement

The University of Washington Human Subjects Review Committee, University of California San Francisco Committee on Human Research, the Kenya Medical Research Institute (KEMRI) National Ethics Review Committee, and ethics review boards at each study site reviewed and approved the study protocol and consent documents.

### Population and Procedures

We used data from a cohort of southern and East African HIV-1 serodiscordant heterosexual couples enrolled in a clinical trial (the Partners in Prevention HSV/HIV Transmission Study) evaluating HSV-2–suppressive therapy with acyclovir 400 mg bid provided to the HIV-1–infected partner to prevent HIV-1 transmission to their HIV-1–seronegative partners. As previously reported, acyclovir decreased plasma HIV-1 levels in the HIV-1–infected partners, but did not reduce HIV-1 transmission risk [19]. The present report is a secondary analysis of data from the subset of 2,236 couples from this prospective cohort in which the HIV-1–infected partner was female [19].

HIV-1–infected partners were required to be seropositive for HSV-2, with a CD4 count ≥250 cells/mm^3^, and without history of AIDS-defining conditions; couples were followed for up to 24 mo. HIV-1–infected women were seen monthly and underwent a pelvic examination at enrollment and every 3 mo to collect a vaginal swab for Gram stain for evaluation of BV. Enrollment vaginal swabs were collected on all participants. Quarterly vaginal swab collection was performed as part of a protocol modification implemented at each site once approved by the site institutional review board; vaginal Gram stain results were not obtained prior to site-specific approval of the protocol modification.

Plasma for HIV-1 RNA quantification was collected at baseline, 3-, 6-, and 12-mo visits, and at study exit; CD4 counts were performed at baseline and every 6 mo. HIV-1–infected partners who met national guidelines for initiation of ART during follow-up were referred to local HIV-1 care clinics, and those who became pregnant were referred to antenatal clinics for prevention of mother-to-child transmission services. HIV-1–infected women underwent a speculum pelvic examination at a visit 6 mo after enrollment, during which an endocervical Dacron swab for HIV-1 RNA quantification was obtained; swabs were not collected at a defined time in the menstrual cycle, although women usually deferred sampling during menstruation. HIV-1–uninfected men were seen quarterly for HIV-1 serologic testing.

Participants received comprehensive HIV-1 prevention including HIV-1 risk-reduction counseling (both individual and as a couple), quarterly sexually transmitted infection (STI) symptom assessment with syndromic treatment of STIs, and provision of free condoms. All participants provided written informed consent.

### Laboratory Methods for Diagnosis of BV

Vaginal swabs collected at enrollment and quarterly follow-up visits were rolled onto glass slides, air dried, and methanol fixed at the study site and subsequently Gram stained at the Center for Microbiology Research Laboratory at KEMRI. Vaginal flora was evaluated using Nugent's criteria [20]: normal vaginal flora, intermediate flora, and BV categories were defined by Nugent's scores of 0–3, 4–6, and 7–10, respectively. Each slide was double-read by two technologists. A digital image of approximately every tenth slide was sent electronically to one of the investigators (CAS) for external quality control (EQC). Our target for concordant results between the laboratory and EQC was ≥90%. A discordant result was defined as a difference in the Nugent's score ≥1, which also caused a change in flora category (e.g., a score difference of 3 to 4, which changes the diagnosis from normal vaginal flora to intermediate flora). External quality control performed on 1,722 (8.7%) of 19,882 slides (the total number of slides included HIV-1–positive women included in this study, and HIV-1–negative women evaluated for a separate analysis) demonstrated an overall concordance of 92.2% (*Κ* = 0.84); the concordance surpassed our predefined value of ≥90% based on expected inter-observer agreement in other studies [20],[21].

### Other Laboratory Procedures

HIV-1 serologic testing was by dual rapid HIV-1 antibody tests performed locally, with positive results confirmed by HIV-1 Western blot at the University of Washington [19]. For couples in which the initially HIV-1–uninfected male partner seroconverted to HIV-1 seropositive, analysis of HIV-1 *env* and *gag* gene sequences from both members of the couple were used to evaluate transmission linkage within the partnership [22]. Serologic testing for HSV-2 and nucleic acid amplification testing for STIs (specifically *Chlamydia trachomatis*, *Neisseria gonorrhoeae*, and *Trichomonas vaginalis*) was done at study enrollment [23]. CD4 quantification was performed using standard flow cytometry. All laboratory procedures followed Good Laboratory Practices, and laboratories were enrolled in External Quality Assessment programs.

HIV-1 RNA was quantified from plasma at baseline, at months 3, 6, and 12, and at study exit; and from the 6-mo endocervical swab specimen (collected at the same visit as the 6-mo plasma specimen) with the COBAS AmpliPrep/COBAS TaqMan real-time HIV-1 RNA assay version 1.0 (Roche Diagnostics). Endocervical swabs were eluted in 1,000 µl of GUSCN lysis buffer, eluted for 15 min, vortexed briefly, and microfuged for 5 s at 14,000*g* to pellet debris before removal of fluid for testing. A final dilution step with 10× PBS was used to achieve sufficient volume for the COBAS AP/TM assay, with a lower limit of quantification of 240 copies (per milliliter for blood plasma and per swab for endocervical samples). Plasma and genital HIV-1 RNA concentrations were log_10_-transformed to approximate normality. Samples below the limit of quantification were assigned values at half that limit.

### Statistical Analysis

The primary outcome was female-to-male HIV-1 transmission, defined as those HIV-1 seroconversion events that were genetically linked within the partnership. Male partners who acquired HIV-1 from an outside partner contributed follow-up time up to HIV-1 seroconversion and were censored thereafter. Follow-up for men was also censored after their HIV-infected partner initiated ART.

The primary exposure was vaginal flora status, as measured at the quarterly study visit prior to each HIV-1 test, in order to represent vaginal flora status during the time of potential HIV-1 exposure to the male partner. If the result at the visit 3 mo prior to HIV-1 testing was expected but missing, the result 6 mo prior was used; if the results at both the 3 and 6 mo prior to HIV-1 testing were expected but missing the period was excluded from analysis. We analyzed vaginal flora in three categories: BV (Nugent score ≥7) and intermediate flora (Nugent score 4–6), each compared with normal flora (Nugent score ≤3). We performed two sensitivity analyses to assess the robustness of our vaginal flora exposure: first, we analyzed vaginal flora at the visit concurrent with HIV-1 serologic testing, and second, we analyzed vaginal flora based on the most severe exposure (highest Nugent category) occurring at either the prior or current visit.

Association between vaginal flora and time-varying covariates was assessed using logistic regression for each of intermediate and BV compared to normal flora, with GEE methods to account for correlation between visits. HIV incidence rates and confidence intervals were computed using Poisson rates; absolute rate differences were calculated [24].

To assess the risk of HIV-infection we performed multivariable Cox proportional hazards analysis to adjust for potential confounding factors, including demographic, medical, and behavioral characteristics. Variables were selected a priori for inclusion based on previously published association with HIV transmission, and included: (1) characteristics from the time of study enrollment: age (of both partners), region (East versus southern Africa), HSV-2 status of the HIV-1 uninfected male partner, male partner circumcision status, trial randomization assignment (acyclovir versus placebo), and laboratory confirmed STIs at enrollment (i.e., *N. gonorrhoeae*, *C. trachomatis*, and *T. vaginalis*) of both partners; and (2) time-dependent variables, including: pregnancy, hormonal contraceptive use, plasma HIV-1 levels, and CD4 count in the female HIV-1–infected partner, genital ulcer disease in both partners, and sexual behavior during the month prior to each visit, as reported by the male HIV-1–uninfected partner (analyzed as any unprotected sexual intercourse with the study partner, any report of outside partners, and total number of sex acts with the study partner). Robust standard errors were used to account for multiple observations from each person in the time-dependent analyses. Differences in plasma and cervical viral load were assessed using linear regression methods, adjusted for repeated observations. Data were analyzed using SAS version 9.2 (SAS Institute Inc.).

## Results

### Study Population

A total of 2,236 couples were included in this analysis (Table 1). The median age of HIV-1–infected female partners was 30 y and the median age of HIV-1–uninfected male partners was 35 y. Most couples were married and cohabitating. Couples engaged in sex a median of four times per month, and 30.5% of couples reported sex that was unprotected by condom use during the month prior to enrollment. Among the HIV-1–infected female participants, the median CD4 count was 481 cells/mm^3^ (interquartile range [IQR] 354–663) and the median plasma HIV-1 RNA concentration was 3.95 log_10_ copies/ml (3.24–4.53).

**Table 1:** Enrollment characteristics, prospective study of 2,236 African HIV-1–seropositive women and their HIV-1 uninfected male partners.

Enrollment Characteristics	Median (IQR) or *n* (%)		
	HIV-1–Infected Female	HIV-1–Uninfected Male	Couple
**Individual characteristics**			
Age, years	30 (25–35)	35 (30–42)	—
Education, years	8 (6–10)	9 (7–12)	—
Hormonal contraceptive use	430 (19.2%)	N/A	—
Male circumcised	N/A	1,228 (54.9%)	—
**Couple characteristics** a			
East Africa (versus southern Africa)	—	—	1,452 (64.9%)
Married	—	—	1,647 (73.7%)
Living together	—	—	1,997 (89.3%)
Years lived together	—	—	5 (2–9)
*n* children together	—	—	1 (0–2)
**Sexual behavior (prior month)** a			
*n* sex acts with study partner	—	—	4 (2–8)
Any unprotected sex with study partner	—	—	682 (30.5%)
Any sex with outside partner	—	—	96 (4.3%)
**HIV-1–seropositive female partner characteristics**			
Plasma HIV-1 RNA, log_10_ copies/ml	3.95 (3.24–4.53)	N/A	—
CD4 count, cells/mm^3^	481 (354–663)	N/A	—
Randomized to acyclovir (versus placebo)	1,108 (49.6%)	N/A	—

a Couple demographic and behavior characteristics as reported by the HIV-1–uninfected man.

N/A, not applicable.

### Follow-up and HIV-1 Incidence

Median follow-up for the HIV-1–seropositive female and HIV-1–seronegative male partners was 20.8 (IQR 15.3–24.1) and 19.3 mo (IQR 13.5–24.0), respectively. Over 3,318 person-years of follow-up, 90 incident HIV-1 infections among men were identified, of which 57 (63.3%) were determined by viral sequencing to be genetically linked within the partnership, for an incidence of linked transmission of 1.72 cases per 100 person-years (95% CI 1.30–2.23). Seven HIV-1 infections occurred in men whose HIV-1–seropositive female partner had no vaginal flora result during the interval when HIV-1 seroconversion occurred. In four of these seven cases, the vaginal swab collection was not expected, while in the remaining three, the result was missing. Thus, 50 HIV-1 incident infections among men with virologically linked HIV-1 transmissions with their female HIV-1–infected partners for whom BV data were available were included in this analysis.

### BV at Baseline and during Follow-up

Of 12,126 visits expected to have vaginal swabs collected during the study, 10,232 (84.4%) had vaginal Gram stain data available. At enrollment, 869 women (41.1%) had BV, 487 (23.0%) had intermediate flora, and 757 (35.8%) had normal vaginal flora. Across all quarterly follow-up visits, the median proportion of women with BV and intermediate vaginal flora was 34.9% (IQR 34.2%–36.3%) and 22.8% (IQR 22.0%–23.9%), respectively, while the median proportion of women with normal vaginal flora was 42.8% (IQR 40.1%–44.1%). Of the 2,221 women with at least one Gram stain result available from the prior 3-mo visit (our main exposure), 337 (15.2%), 113 (5.1%), and 340 (15.3%) had BV, intermediate vaginal flora and normal vaginal flora, respectively, throughout follow-up. An additional 1,151 (51.8%) women had at least a single episode of BV during follow-up.

During follow-up, HIV-1–infected women who had one or more intervals with BV were slightly younger than women who had normal vaginal flora and more likely to have an uncircumcised male partner (Table 2). While periods where unprotected sex was reported did not differ by vaginal flora, HIV-1–infected women with BV were more likely to report an outside sexual partner in the last 30 d than HIV-1–infected women with normal vaginal flora. Plasma HIV-1 RNA concentration was slightly elevated, and mean CD4 count was slightly lower in HIV-1–infected women during intervals with BV in comparison to intervals with normal vaginal flora (Table 2).

**Table 2:** Participant characteristics during quarterly follow-up intervals with BV and intermediate vaginal flora versus normal vaginal flora.

Participant Characteristics	Follow-up Intervals for Analysis of HIV-1 Transmission from Women to Men^a^ (*n* = 2,236 HIV-1–seropositive Women), *n* (%) or Median (IQR)				
	BV Intervals (*n* = 4,025)	*p*-Value^b^	Intermediate Vaginal Flora Intervals (*n* = 2,566)	p-Value^c^	Normal Vaginal Flora Intervals (*n* = 4,474)
**Demographic characteristics**					
Age of HIV-1–seronegative partner, years	35 (29–42)	0.02	36 (30–43)	0.53	36 (30–42)
Children within the partnership					
Having at least one child within the partnership	2,405 (59.8%)	<0.0001	1,815 (70.7%)	0.10	3,264 (73.0%)
**Sexual behavior, HIV-1 uninfected partner (past month)**					
Any unprotected sex with study partner	432 (10.7%)	0.88	267 (10.4%)	0.85	474 (10.6%)
Any sex with an outside partner	608 (15.1%)	0.19	300 (11.7%)	0.13	465 (10.4%)
Male circumcision	2,158 (53.6%)	<0.001	1,519 (59.2%)	0.50	2,697 (60.3%)
Number of sex acts with study partner	3 (1–6)	0.98	3 (2–6)	0.91	3 (2–6)
**Clinical characteristics**					
CD4 count (cells/mm^3^) in the HIV-1–infected partner	453 (329–624)	0.82	447 (319–631)	0.07	464 (342–654)
Plasma HIV-1 level (log_10_ copies/ml) in the HIV-1–infected partner	4.09 (3.27–4.69)	<0.0001	4.01 (3.25–4.68)	0.001	3.88 (3.11–4.53)
HSV-2 serostatus, male partner	2,438 (60.7%)	0.32	1,554 (60.6%)	0.20	2,619 (58.6%)
Genital ulcer disease, HIV-1–infected partner on exam	92 (2.6%)	0.07	43 (1.9%)	0.81	71 (1.8%)
Genital ulcer disease, HIV-1–seronegative partner	36 (0.9%)	0.358	12 (0.5%)	0.40	30 (0.7%)
Pregnant, female partner (current visit)	263 (6.8%)	0.68	174 (7%)	0.40	348 (8.1%)

a Comparisons between BV exposure groups are adjusted for correlation by multiple measures from the same participant using generalized estimating equations. The number of data points assessed for each cell is total number of visits with each covariate characteristic during study follow-up.

b Comparison of BV intervals to normal vaginal flora interval.

c Comparison of intermediate vaginal flora intervals to normal vaginal flora intervals.

### Effect of BV on Incidence of Female-to-Male HIV Transmission

During the study, HIV-1 incidence was 2.91, 1.48, and 0.76 per 100 person-years in men whose female partner in the seroconversion interval had BV, intermediate vaginal flora, and normal vaginal flora, respectively (Table 3). In unadjusted analysis, BV was associated with a 3.62-fold increased risk (95% CI 1.74–7.52) and a 2.15 increased attributable rate (95% CI 1.04–3.25) per 100 person-years of female-to-male HIV-1 transmission in comparison to women with normal vaginal flora (Table 3). In multivariable analysis controlling for sociodemographic factors, sexual behavior, male circumcision, sexually transmitted infections, pregnancy, and plasma HIV-1 RNA in female partners, men whose HIV-1–infected female partners had BV 3 mo prior to identifying HIV-1 seroconversion had a 3.17-fold increased adjusted risk (95% CI 1.37–7.33) of female-to-male HIV-1 transmission. Intermediate flora in comparison to normal vaginal flora was not associated with an altered risk of female-to-male HIV-1 transmission (Table 4). The two sensitivity analyses looking at vaginal flora at the same visit as HIV-1 seroconversion detection and the highest category of vaginal flora between the prior and same visit as HIV-1 seroconversion detection were consistent with these results (Table 4). Lastly, we did not find evidence of an effect of male circumcision on the relationship between BV in the HIV-1–seropositive female partner and linked HIV-1 infections in men (unpublished data).

**Table 3:** Incidence of HIV-1 transmission to men, by the vaginal flora category of their female HIV-1–infected partner.

Female-to-Male HIV-1 Transmission in Relation to Vaginal Flora^a^	HIV-1 Cases	Person-Years of Follow-up	HIV-1 Incidence (per 100 Person-years) (95% CI)	Absolute Risk Difference (95% CI)
All seroconversions	57	3,317.8	1.72 (1.30–2.23)	—
Seroconversions with missing BV status	7	—	—	—
Seroconversions with BV status	50	2,924.1	1.71 (1.27–2.25)	—
Normal vaginal flora (Nugent score 0–3)	9	1,181.6	0.76 (0.35–1.45)	ref
Abnormal vaginal flora (Nugent score 4–10)	41	1,742.5	2.35 (1.69–3.19)	1.59 (0.63–2.56)
Intermediate vaginal flora (Nugent score 4–6)	10	676.4	1.48 (0.71–2.72)	0.72 (−0.24 to 1.67)
BV (Nugent score 7–10)	31	1,066.1	2.91 (1.98–4.13)	2.15 (1.04–3.25)

a For the primary analytic approach, vaginal flora from the adjacent previous visit (usually 3 mo prior) was used. If this result was missing, the most recent vaginal flora result from the visit 6 mo prior was used; otherwise the result was considered missing.

**Table 4:** Risk of female-to-male HIV-1 transmission among men whose HIV-1–infected female partners had BV and intermediate vaginal flora in comparison to men whose HIV-1–infected female partners had normal vaginal flora.

Model	Variable Categories	HR, 95% CI	AHR^a^, 95% CI
**Primary analysis**			
Pre-visit BV status	BV versus normal vaginal flora	3.62 (1.74–7.52)	3.17 (1.37–7.33)
	Intermediate versus normal vaginal flora	1.88 (0.77–4.6)	1.63 (0.62–4.26)
**Sensitivity analyses**			
Current-visit BV status	BV versus normal vaginal flora	5.30 (2.21–12.74)	4.17 (1.74–9.97)
	Intermediate versus normal vaginal flora	2.00 (0.67–5.95)	1.73 (0.57–5.19)
More severe BV status between pre- and current-visit	BV versus normal vaginal flora	7.19 (2.59–19.94)	6.96 (2.11–22.98)
	Intermediate versus normal vaginal flora	2.28 (0.67–7.76)	2.14 (0.53–8.60)

a Multivariable Cox proportional hazards analysis (adjusted hazard ratio [AHR]), adjusting for the following: Fixed covariates: age, geographic region (southern Africa versus Eastern Africa), HSV-2 status of male partner at study enrollment, male circumcision, randomization treatment assignment, and both female and male sexual transmitted disease at study enrollment (laboratory confirmed gonorrhea, chlamydia, and trichomonas); Time-dependent covariates (per quarterly visit): pregnancy (current visit), hormonal contraception (current visit), plasma HIV-1 viral load of female partner (from enrollment and month 3, 6, 12, and study exit), unprotected sex act with study partner (current visit) (based on reporting from male partner), CD4 count of female partner (6 monthly), outside partners (current visit, based on reporting from male partner), number of sex acts with their study partner (current visit), and genital ulcer disease (current visit) (based on physical exam from both partners).

### Effect of BV on Genital and Plasma HIV-1 RNA Concentration

The mean log_10_ concentration of HIV-1 RNA in genital secretions and plasma were slightly elevated in participants with intermediate vaginal flora (log_10_ difference: 0.21 and 0.16, respectively) and BV (log_10_ difference: 0.19 and 0.18, respectively) (Table 5). After controlling for plasma HIV-1 RNA concentration at the same 6-mo visit, the mean log_10_ concentration of HIV-1 RNA in genital secretions was no longer significantly associated with vaginal flora (Table 5).

**Table 5:** Log_10_ HIV-RNA concentration in plasma (baseline and follow-up) and female genital secretions (6-mo follow-up visit) compared by vaginal flora category (normal vaginal flora, intermediate vaginal flora, and BV).

Vaginal Flora	*Log_10_ HIV-RNA Concentration (Mean ± SD)*	*p-Value Versus Normal Vaginal Flora*	*p-Value Versus Normal Vaginal Flora* *
**Genital HIV-1 RNA**			
Normal vaginal flora	3.04±0.99	ref	—
Intermediate vaginal flora	3.25±1.01	0.0035	0.058
BV	3.23±0.99	0.0023	0.095
**Plasma HIV-1 RNA**			
Normal vaginal flora	3.81±1.00	ref	—
Intermediate vaginal flora	3.96±1.07	0.037	—
BV	3.99±1.07	0.0056	—

The lower limit of quantification was 240 copies/ml for blood fluid and 240 copies/swab for endocervical samples.

*: After adjusting for log_10_ plasma HIV-1 RNA concentration.

SD, standard deviation.

## Discussion

In this prospective study of more than 2,200 southern and East African HIV-1–seropositive women and their HIV-1–seronegative male partners with genetic linkage of HIV-1 transmission pairs, we found that BV was independently associated with a 3-fold increased risk of female-to-male HIV-1 transmission. The potential significance of this finding is substantial, given that 35%, 15%, and 52% of women in this study had BV at enrollment, throughout follow-up, and at least one interval of BV during the 2 y of follow-up, respectively. This proportion of HIV-1–seropositive women with BV is consistent with prior studies demonstrating a prevalence of BV ranging from 30%–55% among women in sub-Saharan Africa [8]–[10]. Thus, assuming that the association we report is causal, BV may account for a substantial population attributable risk percent for new HIV-1 infections in men in Africa.

Although genital HIV-1 RNA predicts female-to-male HIV-1 transmission independent of the HIV-1 RNA concentration in blood [25], we found only a modest (0.2 log_10_) increase in HIV-1 RNA in women with BV in comparison to those with normal vaginal flora. Thus, it is likely that increased genital HIV-1 RNA caused by BV only partially explains our results. Most cross-sectional and longitudinal studies have found that women with BV have higher concentrations of HIV-1 RNA in genital secretions in comparison to women with normal vaginal flora [12],[13],[26]. However, two prospective studies did not find differences in genital HIV-1 RNA concentration associated with BV [14],[27]. Differences with previous studies that found an association between BV and genital HIV-1 shedding could be due to the proportion of women with BV who were symptomatic, with potentially higher levels of inflammation associated with symptomatic BV, and the short duration (i.e., 14 d) after BV treatment in which vaginal samples were collected in the longitudinal studies to measure genital HIV-1 RNA, which may have been too soon for decreased T-cell activation [18], proinflammatory cytokines [28], or reestablishment of lactobacilli predominant flora [29].

An additional hypothesis to explain our findings is that BV in a female partner may indirectly increase HIV-1 susceptibility in men. A growing body of evidence suggests that the female and male genital microbiota is shared between sexual partners [8],[29],[30]. Recent data from Uganda have demonstrated that male circumcision reduces the risk of BV in female partners and that bacterial flora associated with BV commonly colonize the penis including the distal urethra [30],[31]. Anaerobic and other bacteria increased in male partners of women with BV may cause inflammation by activating Langerhans cells and CD4+ T-cells, thereby increasing target cells for HIV-1 and susceptibility to HIV-1 infection [32],[33]. Interestingly, male circumcision did not affect or modify the relationship between BV and female-to-male HIV-1 transmission in our study. In comparison to pre-circumcision abundances of bacterial phylotypes, post-circumcision abundances of anaerobic bacteria decreased, while abundances of facultative anaerobic bacteria increased significantly in the Rakai study [31]. Potential mechanisms need investigation, including whether the male genital microbiota, in particular anaerobes, are associated with urethral and penile inflammation and activation of Langerhans cells and CD4+ T-cells, which could increase risk of HIV-1 infection in men.

Lower socioeconomic status has been associated with higher BV prevalence [8],[34]. Previous studies have also implicated race, multiple sex partners, trichomoniasis, HIV-1 infection, intrauterine device use, vaginal douching, recent antibiotic use, and the absence of vaginal colonization by H_2_O_2_-producing lactobacilli as risk factors for BV [8],[9],[34]–[38]. Following treatment, BV clinically recurs in 20%–30% of women within 3 mo [39], and recurs in approximately 75% of women with symptomatic as well as asymptomatic BV within 2 mo of treatment [29]. One reason for the high prevalence of BV and its frequent recurrence may result from the transfer of potentially pathogenic bacteria between heterosexual partners through genital, and potentially orogenital contact [40],[41].

The high prevalence and frequent recurrence of BV has led to the development of several new strategies including frequent presumptive antibiotic treatment [42], and use of probiotic lactobacilli as an adjuvant or an alternative to antimicrobial therapy [43]–[45]. Recent advances in understanding the microbiota associated with BV [6],[7], including the ability of *G. vaginalis* and to a lesser degree *Atopobium vaginae* to form biofilms recalcitrant to antibiotic treatment [46],[47], may eventually lead to therapies that maintain a lactobacilli-predominant flora in the vagina.

Our study has several strengths starting with its large and diverse population of HIV-1–serodiscordant couples recruited from across multiple sites in southern and East Africa. Furthermore, genetic linkage of female-to-male transmitted HIV-1 minimized misclassification in our analysis [22]. One limitation of our analysis is that we do not know the specific vaginal flora present at the time of HIV-1 infection since vaginal microbiota can fluctuate weekly [48]. To address this, we conducted two sensitivity analyses, the first evaluating vaginal Gram stain results at the same visit when HIV-1 seroconversion was first noted, and the second evaluating the severity of vaginal flora between that and the prior visit. Both evaluations confirmed the results of our primary analysis. The cohort was a highly selective population (e.g., all participants underwent couples HIV counseling and testing, enrolled in an HIV-1 prevention randomized clinical trial, and index participants had a CD4 count ≥250 cells/mm^3^ at enrollment), which could impact on the generalizability of our results. Furthermore, all HIV-1–infected partners were co-infected with HSV-2; however, HSV-2 seroprevalence is >80% among HIV-1–infected persons in sub-Saharan Africa [19] and thus is unlikely to limit the generalizability of our findings. In addition, the relatively small number of female-to-male HIV-1 transmissions (nine among women with normal vaginal flora versus 31 among women with BV) requires mention. Finally, residual or unmeasured confounding, which cannot be completely excluded, could affect the significance of our findings.

This study clearly demonstrates that BV is associated with an increased risk of female-to-male HIV-1 transmission. BV is a highly prevalent condition among HIV-1–infected women. The association of BV with increased infectiousness of HIV-1–infected women requires additional research to understand potential pathogenic mechanisms as well as the etiology, treatment, and prevention of BV. While a large community randomized controlled trial that provided presumptive treatment of STIs including metronidazole for BV failed to reduce HIV-1 incidence [49], ongoing studies are evaluating more frequent presumptive BV therapy [42], while others are studying naturally occurring and genetically enhanced probiotics to reduce recurrent BV [44],[45],[50],[51]. A lactobacillus-predominant vaginal flora might not only reduce the risk of HIV-1 acquisition in women [9],[11], but also HIV-1 transmission to male partners, and points to the potential benefits of using the human microbiota to prevent disease.

## Abbreviations

ART: antiretroviral therapy
BV: bacterial vaginosis
HSV: herpes simplex virus
IQR: interquartile range
STI: sexually transmitted infection
